# Oscillations and Episodic Memory: Addressing the Synchronization/Desynchronization Conundrum

**DOI:** 10.1016/j.tins.2015.11.004

**Published:** 2016-01

**Authors:** Simon Hanslmayr, Bernhard P. Staresina, Howard Bowman

**Affiliations:** 1 University of Birmingham, School of Psychology, Birmingham, UK; 2 University of Kent, School of Computing, Canterbury, UK

**Keywords:** episodic memory, oscillations, cross-frequency coupling, alpha/beta, theta, gamma, hippocampus, MTL, complementary learning systems

## Abstract

Brain oscillations are one of the core mechanisms underlying episodic memory. However, while some studies highlight the role of synchronized oscillatory activity, others highlight the role of desynchronized activity. We here describe a framework to resolve this conundrum and integrate these two opposing oscillatory behaviors. Specifically, we argue that the synchronization and desynchronization reflect a division of labor between a hippocampal and a neocortical system, respectively. We describe a novel oscillatory framework that integrates synchronization and desynchronization mechanisms to explain how the two systems interact in the service of episodic memory.

## Trends

Data from rodent as well as human studies suggest that theta/gamma synchronization in the hippocampus (i.e., theta phase to gamma power cross-frequency coupling) mediates the binding of different elements in episodic memory.

*In vivo* and *in vitro* animal studies suggest that theta provides selective time windows for fast-acting synaptic modifications and recent computational models have implemented these mechanisms to explain human memory formation and retrieval.

Recent data from human experiments suggest that low-frequency power decreases in the neocortex, most evident in the alpha/beta frequency range, mediate encoding and reinstatement of episodic memories.

The content of reinstated memories can be decoded from cortical low-frequency patterns.

## The Synchronization, Desynchronization Conundrum

Brain **oscillations** (see Glossary) provide temporal windows for neural firing and shape synaptic plasticity by **synchronizing** and **desynchronizing** neural assemblies. Oscillations therefore have a particularly high potential to shed light on the mechanisms underlying episodic memory 1, 2. However, over the past few years a conundrum has emerged regarding how brain oscillations relate to memory [3]. Whereas some studies highlight the role of synchronized activity, mainly in the theta (∼3–8 Hz) and gamma (∼40–80 Hz) frequencies [1], others highlight the role of desynchronized activity, mainly in the lower-frequency ranges (<20 Hz [4]). This conundrum exists at both an empirical and a theoretical level. Based on Hebb's seminal idea – neurons that fire together, wire together – a strong case can be made that synchrony is required for memory formation 1, 2. However, mathematical information theory, alongside physiological studies in animals, postulates that high synchrony, especially in the lower-frequency ranges (<20 Hz), reduces information coding 4, 5, 6. We here aim to integrate these two seemingly incompatible concepts and present a mechanistic framework to resolve this conundrum. Building on complementary learning systems [7], we assume a division of labor between a hippocampal system, which mainly binds information, and a neocortical system, which mainly represents the content of this **information**. Based on recent findings, we argue that this division of labor is visible in the two opposing oscillatory behaviors, with the hippocampal system showing synchronization in the theta/gamma range 8, 9, 10, mediating binding [11], and the neocortex showing low-frequency desynchronization [4], mediating the representation of information 12, 13.

## Hippocampal Synchronization in the Service of Episodic Memory

The critical role of the hippocampus for intact episodic memory has been firmly established by neuropsychological studies, animal models, computational models, as well as human neuroimaging [14]. Notably, hippocampal engagement seems particularly important for the key characteristic of episodic memories; that is, binding discontiguous (i.e., separate) event details into rich associative memory traces [15]. How is this feat accomplished mechanistically? Gamma oscillations, operating at timescales below 30 ms, have recently been proposed as the prime candidate to facilitate learning-related synaptic changes such as spike-timing-dependent plasticity (STDP)/long-term potentiation (LTP) [1]. Does the hippocampus recruit such gamma oscillations in the service of episodic memory formation and retrieval?

Direct hippocampal recordings from human epilepsy patients have found that gamma power increases correlate with both successful memory encoding [16] and retrieval [17]. One caveat related to these results is that, on closer examination, the gamma effects were found to be part of more broadband power increases, ranging from 30 Hz to 100 Hz 18, 19. The functional significance of such broadband power increases remains under debate, but consensus has emerged that they reflect multi-unit spiking activity rather than true oscillatory activity (20, 21, but see [22]). However, recent recordings from the primate hippocampus have succeeded in showing narrow-band gamma power increases as well as spike-field coherence between single units and gamma-band LFPs during successful learning [23].

**Figure 1:**
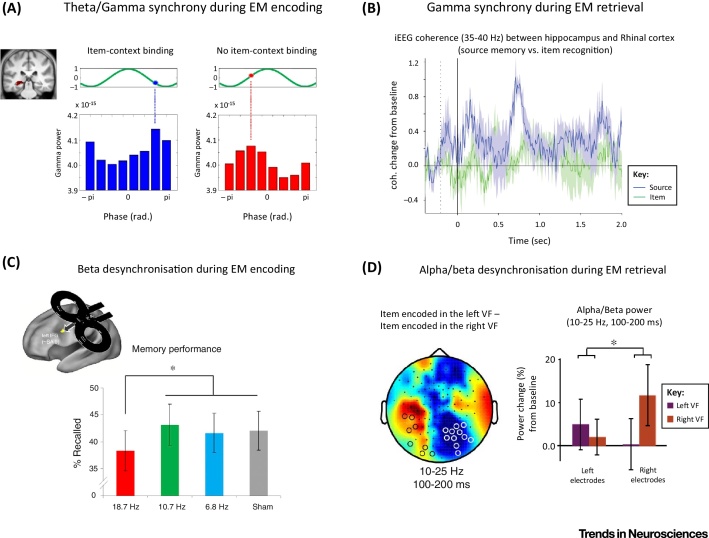
Studies Showing Hippocampal Theta/Gamma Synchronization and Alpha/Beta Desynchronization During Encoding and Retrieval of Episodic Memories (EMs). (A) Gamma power coupled to different phases of theta predicts whether item–context binding occurs or does not occur, as tested in a subsequent memory paradigm where the contextual overlap between encoding and retrieval was directly manipulated [11]. (B) Results from an intracranial electroencephalography (EEG) study showing gamma phase synchronization between the hippocampus and rhinal cortex during successful associative recognition (i.e., source memory) compared with simple item recognition. (C) Preventing beta desynchronization (i.e., 18.7-Hz stimulation) at the left inferior frontal cortex via rhythmic transcranial magnetic stimulation (rTMS) selectively impairs memory encoding. (D) Alpha/beta power decreases during retrieval indicate the visual field (VF) where a stimulus was initially encoded (i.e., alpha/beta power decreases indicate memory reactivation). (A,B,C,D) reproduced and modified with permission from 11, 25, 39, 47, respectively.

Perhaps even more compelling evidence for hippocampal gamma oscillations supporting episodic memory processes comes from studies examining short-range inter-regional synchronization, particularly between the hippocampus and the adjacent entorhinal cortex (EC). In human epilepsy patients, gamma-band coherence has been found to increase between these regions as a function of successful episodic encoding [24] as well as retrieval [25] (Figure 1B). Recordings from rodent models have gone further to identify a frequency-specific ‘routing function’ of gamma oscillations, such that the CA1 subregion is functionally coupled with CA3 versus the EC in different gamma sub-bands [26]. Establishing the relevance of this coupling for learning processes, a recent study has linked the gamma-band coherence between CA1 and the EC to the encoding and retrieval of odor/space associations [27].

In summary, converging evidence has accumulated across different species and across different experimental paradigms for a critical role of synchronized hippocampal gamma oscillations in the service of episodic memory. The pressing question is then how these oscillations are themselves regulated. Ignited by the hallmark observation of theta phase precession of hippocampal place cells [28], much research has been dedicated to understanding the role of the theta rhythm in orchestrating memory-related neural signals [29]. A striking example is the finding – observed via direct recordings from the human hippocampus – that successful episodic encoding may not necessarily rely on the net increase in hippocampal firing rates but on the temporal precision of single-unit firing with respect to the concurrent theta phase [8].

Importantly, recent research has begun to investigate not only how the theta rhythm may clock single-unit firing but also how oscillatory patterns in the gamma range may be related to the ongoing theta phase [9]. For instance, the abovementioned coupling between CA1 and CA3 versus the EC was not only expressed in different gamma sub-bands but also occurred at different phases of the theta cycle [26]. Moreover (and notwithstanding the abovementioned question of whether gamma power increases reflect true oscillatory changes versus multi-unit spiking), evidence has accumulated in both rodent models and human intracranial recordings that hippocampal theta–gamma coupling is linked to successful episodic memory processes [30]. Of particular importance is a recent magnetoencephalography (MEG) study that showed that item–context binding, a hallmark of episodic memory, critically depends on theta–gamma coupling in the medial temporal lobe (MTL), and specifically on whether gamma oscillations are coupled to the peak or the trough of a theta cycle (Figure 1A) 11, 30, 31. Notably, the frequency range showing the strongest modulatory effects is slightly lower in humans (∼3–4 Hz) than in rodents (∼8 Hz), in line with the notion that functionally homologous oscillations occur at increasingly slower frequencies as brain size increases across species 32, 33.

Taking these findings together, the mnemonic functions of the hippocampus appear to be intimately linked to neuronal synchronization in the gamma frequency band, with a regulatory influence of the phase of ongoing theta oscillations (Figure 1A,B).

## Cortical Desynchronization in the Service of Episodic Memory

Oscillatory power decreases during the formation of memories (i.e., encoding) are typically observed in the lower-frequency ranges (<20 Hz), especially during encoding of items that are later remembered compared with later not remembered 34, 16, 35, 36, 37. For instance, during encoding of verbal material, alpha/beta power decreases (∼12–18 Hz) are most evident in the left inferior prefrontal cortex 16, 37, 38. Importantly, such left frontal beta power decreases are not a simple (incidental) byproduct of memory formation but are causally relevant, as demonstrated in a recent combined electroencephalography (EEG)–rhythmic transcranial magnetic stimulation (rTMS) study [39] (Figure 1C). Given that the left inferior prefrontal cortex is strongly involved in semantic processing in general, these results fit with several findings in the language domain showing that left prefrontal beta power decreases during semantic processing 40, 41. Memory formation for nonverbal material (i.e., images) is also accompanied by power decreases; however, these decreases apparently occur more in the alpha range at parieto-occipital regions [34]. Clearly, more studies are needed to investigate alpha/beta power decreases during memory formation of different materials (including words, images, and sounds), but the pattern of results so far suggests that alpha/beta desynchronization indexes information processing in specialized cortical modules during the perception of an event 42, 43 and therefore predict its likelihood of being later remembered. Of note, this negative relationship between power decreases and memory formation might also extend to the theta frequency range, as indexed by several recent studies showing that theta power decreases correlate with memory formation 16, 44.

A similar picture arises for power decreases during memory retrieval. For instance, the topography of alpha/beta power decreases varies with the type of retrieved material (i.e., words versus faces [45] or locations versus objects [46]), suggesting that power decreases indicate material-specific memory reactivation. Direct support for this comes from studies that presented objects during encoding to either the left or right visual hemifield and showed that memory retrieval of centrally presented objects is indeed reflected by alpha/beta power decreases contralateral to the site at which the item was encoded 13, 47 (Figure 1D). Using multivariate pattern analysis of time–frequency data, two MEG/intracranial EEG (iEEG) studies went one step further and demonstrated that the content of reactivated material can be reliably decoded from alpha/beta frequencies 12, 48. For instance, applying a temporal-pattern-analysis approach, Staudigl *et al.* [12] demonstrated that the reactivation of individual dynamic contexts (i.e., movie clips) can be decoded from the temporal pattern of beta phase in material-specific cortical areas (i.e., parahippocampal area, visual cortex; [12]). Together, these results offer an interesting possibility for the mechanistic role of low-frequency power decreases: decreases in oscillatory activity enable a neural assembly to express a stimulus-specific code by allowing a more complex (i.e., information-rich) temporal phase trajectory. Arguably, such a coding mechanism would not work very efficiently in situations of high synchrony (i.e., a stationary signal), where large populations of neurons are entrained to the same rhythm [4].

Taking these findings together, we postulate that low-frequency power decreases reflect the active engagement of cortical modules during encoding and retrieval of memories. An open question is what a low-frequency power decrease means mechanistically (i.e., at the neural level) and how it relates to the theta/gamma dynamics in the hippocampal system. Although more research is needed to answer this complex question, a convergent picture can be derived from three different recent frameworks. (i) Following the alpha inhibition framework, low-frequency power decreases could act as a gating mechanism whereby decreasing alpha power increases firing rates 42, 43. Notably, this negative relationship between oscillatory activity and neural firing might also extend to higher (beta) and lower (theta) frequencies [9]. (ii) A second line of research suggests that synchronized firing in low frequencies is a main contributor to the trial-by-trial variance in neural firing (termed ‘**noise correlations**’), thereby reducing the reliability with which a neural code is expressed in a population of neurons 6, 49. Decreasing the amplitude of these low-frequency oscillations would reduce such neural noise correlations and thus increase the reliability of a neural code that is conveyed to downstream neurons (i.e., in the hippocampus). (iii) Finally, power decreases themselves could be a mechanism to de-correlate neural activity to enhance the neural coding capacity *per se*. Power decreases might thereby allow flexible phase adjustments in a neural population to form a temporal code representing the identity of a specific stimulus 4, 12, 50.

**Figure 2:**
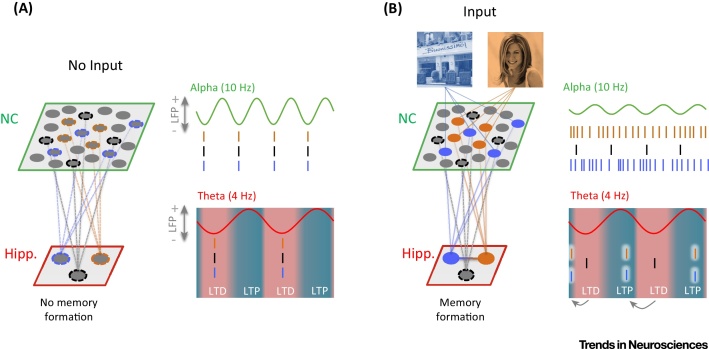
A Schematic of the Current Framework. (A) In the absence of external input, high alpha power is evident in the neocortex (NC), with NC neurons firing at a low rate. In this state, the NC neurons have little impact on their downstream neurons in the hippocampus. long-term potentiation (LTP)/long-term depression (LTD) is regulated by the theta phase, whereby, in the absence of input, the synaptic connections become weakened (LTD). (B) Input reduces the (effective) firing threshold for stimulation-specific populations in the NC, which, in this case, respond to two stimuli. On the population level, neuronal firing increases, which leads to a decrease in synchrony and alpha power. The increased firing in the two NC populations of neurons would in turn reduce the (effective) firing threshold of their hippocampal stimulation-specific downstream neurons, causing phase precession (indicated by arrows) and hence LTP, which then forms an association between the two stimuli. Note that the phase of theta changes depending on which hippocampal subregion it is recorded from [29], being 180^o^ phase shifted between the hippocampal fissure and CA1/CA3. We here plot theta as would be recorded in CA1 (and CA3), in keeping with studies that showed LTP to occur at the peak (+) and LTD to occur at the trough (−) of theta 53, 55. Moreover, as presented here, the peak of theta is in fact the functionally inhibitory phase, while its trough is the functionally excitatory phase.

The common notion between all of these different frameworks, however, is that power decreases enable a neural assembly to express a neural code in some form; that is, via an increase in firing rate, a reduction in noise, or phase encoding. Any such code would be meaningless if it were not interpreted by a ‘reader’ [2]. Here we assume that this reader is the hippocampus (Figure 2).

## Reconciliation: A Synchronized Hippocampus and a Desynchronized Cortex

The **Complementary Learning Systems (CLS)** theory has championed the position that the neocortex and hippocampus provide different but complementary representational formats, with the neocortex supporting a rich integrated representation learned over many experiences and the hippocampus providing a sparse representation learned in ‘one shot’ (or at least few shots). The theory also proposes that the hippocampus, sitting at the end of the processing pathway, provides online learning of conjunctive representations, which bind the constituent elements of an episodic memory. This is implemented by strengthening the synapses between contributing neurons through LTP.

We argue that the CLS theory provides a framework within which the disparity in synchrony of oscillations between cortex and hippocampus can be reconciled. Stated explicitly, we propose that the complementarity of component subsystems is reflected in the disparity of oscillatory dynamics that these subsystems exhibit. Our proposal is most clearly understood by considering the sequence of steps that we envisage realize encoding of episodic memories (illustrated in Figure 2). (i) Sensory stimulation (e.g., seeing a person in a particular place) induces a reduction in alpha/beta oscillations to allow neocortical units to encode the content of that stimulation 42, 43 by pushing their firing rates up significantly. Whether alpha/beta power reductions drive an increase in neural firing or whether an increase in neural firing drives a reduction in alpha/beta power is an open question; however, the net result is in both cases an observable decrease of alpha/beta power together with increased and desynchronized firing of broadly tuned neurons. (ii) This stimulation-specific firing rate increase in the neocortex in turn drives corresponding stimulation-specific hippocampal units to move their firing forward in the phase of theta. In the case where hippocampal units are not receiving stimulus-specific drive from neocortical units, they would fire in the excitatory phase of theta; that is, in the trough of theta as recorded in CA1/CA3 [51] (Figure 2A). In the case of stimulation (i.e., encoding), those units that respond to the presented stimuli would advance their spiking with respect to the ongoing theta oscillation, showing a pattern that is similar to the well-documented phenomenon of phase precession [28]. That is, the increased external driving excitation enables the stimulation-specific hippocampal units to hit their firing threshold earlier in the theta phase; that is, closer to the inhibitory phase of theta (i.e., the peak in CA1/CA3) – a time point where most hippocampal units are silent 51, 52. (iii) This advancement initiates a temporal segregation in which precessed units come to fire in an earlier and separate gamma cycle episode. This again takes inspiration from phase precession, whereby precession specifically advances the gamma cycle of a volley of spikes in the phase of theta. Importantly, we propose that the segregation of stimulation-sensitive from non-sensitive units enables LTP to be selectively applied. Thereby, the earlier phase of theta that stimulation-specific hippocampal units are driven to fire in is specifically one in which LTP occurs. See Box 1 for possible mechanisms that could underlie this increase in LTP. (iv) This increased segregation into gamma cycles would explain how the coupling of theta phase and gamma power would correlate with memory formation and item–context binding in the hippocampus 11, 30, 31. (v) As a result of this selective LTP, stimulation-specific units would become strongly interconnected in the hippocampus, laying down a memory of the episode. Following CLS theory, these associative connections would facilitate retrieval through completion of a partial stimulation pattern. Importantly, the sparse, pattern-separated nature of the hippocampal representations ensures that only a small proportion of the entire population of units would precess forward in the theta oscillation, which in turn ensures that learning is selective.

The observation central to the conundrum we seek to resolve, that gamma synchronizes to theta in the hippocampus during episodic memory encoding, is critical to the phase advancement argument just made. That is, the specific presence of strong gamma-to-theta synchrony provides the mechanism by which firing of driven units steps out and forward in the theta cycle, enabling relevant synapses to be selectively subject to LTP. In this sense, one could argue that gamma-to-theta synchronization is key to the distinctive learning capacity of the hippocampus while alpha/beta desychronization is key to the neocortex's capacity to represent information.

## Caveats and Links to Other Memory Models/Theories

One seemingly counterintuitive consequence of our framework, and of the animal work it builds on 53, 54, 55, is that both LTP and long-term depression (LTD) (i.e., learning and forgetting) occur sequentially in an alternating manner depending on theta phase. This would be a problem for our framework if the amount of the positive weight change at any single synapse during LTP were equal to the amount of the negative weight change at a synapse during LTD. However, this is unlikely to be the case. In our framework, LTD occurs for synapses of ‘irrelevant’ units; that is, units that do not currently receive input from their cortical counterparts and therefore fire during the theta trough (as recorded in CA1/CA3). Notably, most hippocampal pyramidal cells are active during that time [51]; consequently, the actual weight change that is applied to these units can be assumed to be fairly small (Box 1) [56]. Thus, our framework predicts a weak amount of forgetting over time, especially for units that are not reactivated, which resonates well with the fact that memories fade over time. Moreover, this assumption is also in line with a plethora of findings showing that reactivation of memories during wakefulness [57] and sleep [58] counteracts this forgetting. This aspect of our framework also resonates well with recent ideas highlighting the role of forgetting as a highly organized process that keeps our memory system flexible, goal oriented, and organized 59, 60.

In the presented framework, we focused on the interaction between two of the most ubiquitous oscillatory dynamics in the brain, theta oscillations in the hippocampus and alpha/beta oscillations in the neocortex. Therefore we did not include a detailed description of how theta oscillations regulate encoding and retrieval dynamics within the different hippocampal subfields. These mechanisms are described in great detail in other theoretical papers (e.g., [29]) and have been implemented in recent computational models [52]. Notably, although the exact mechanisms of how LTP/LTD and theta phase precession are implemented differ slightly, the basic characteristics of our framework and these previous studies are very well in line with each other. In general, it would be helpful if more computational memory models take into account the intrinsic oscillatory behavior of the brain to relate the electrophysiological patterns to memory processes. Similarly, our understanding of the mechanisms that neurally drive desynchronization in the cortex during memory formation is rather limited to date. Previous animal studies in the attention [61] and motor [62] domain suggest possible candidate mechanisms (i.e., thalamocortical interactions), but whether these can be extended to memory remains to be investigated.

As for any theoretical framework, there are potential limitations and challenges that need to be considered. For instance, no human data currently exist that supports that hippocampal theta oscillations regulate LTP/LTD in a similar way as they do in rodents 53, 54, 55. Similarly, although place cells [63] and grid cells [64] have been discovered recently in humans, no study has yet shown the phenomenon of phase precession in the human hippocampus. Any mechanistic framework that aims to explain episodic memory needs to address how synaptic modifications occur at a rapid timescale. The theta-mediated LTP/LTD mechanisms observed in animals provide a mechanism that fulfils these criteria. However, STDP (Box 1), which, it has been argued, operates on longer timescales, might be a more questionable mechanism for episodic memory formation. Interestingly, animal studies suggest that for STDP protocols to reliably induce LTP within a few seconds the stimulation has to occur in the theta rhythm [65], suggesting an interaction between STDP and theta oscillations, which would then be in line with the framework proposed here.

## Concluding Remarks

The aim of this opinion article was to provide a mechanistic framework within which we can understand the different roles of neural synchronization and desynchronization in the service of episodic memories. Linking findings from the recent electrophysiological literature and investigations of oscillatory correlates of episodic memory formation and retrieval with the well-known CLS framework, we argue that hippocampal theta/gamma synchronization is necessary for binding episodes whereas cortical low-frequency desynchronization is necessary to represent the content of these episodes. This framework makes several clear predictions (Box 2) and raises important open questions (see Outstanding Questions) that should be tested in the future.

Outstanding Questions What are the neural mechanisms underlying memory-related low-frequency power decreases?

Is hippocampal theta/gamma synchronization directly related to cortical desynchronization as reflected in alpha/beta power decreases?

Does cortical desynchronization support the representation (i.e., reinstatement) of memories?

Is theta phase precession in hippocampal areas CA1 and C3 related to cortical low-frequency power decreases?

Does phase precession occur in the human hippocampus and does it separate stimulation-specific from non-stimulation-specific units?

Can a computational model that implements the here-proposed hippocampal synchronization and neocortical desynchronization mechanisms account for experimental data?

## Box 1 Mechanisms for Theta-Phase-Dependent Synaptic Modifications

We envisage three possible ways in which advancement of firing in the phase of theta could induce increased LTP.

There is considerable evidence that the strength of LTP in the hippocampus (or indeed whether there is LTP or LTD) varies with the phase of theta 53, 54, 55. *In vivo* [55] as well as *in vitro* [53] studies showed that stimulation during the positive peak of theta favors LTP. Notably, this positive theta peak in CA1 is effectively the hyperpolarizing phase (i.e., inhibitory for most hippocampal pyramidal cells 29, 51). Together, these results fit with our model, where cortically driven neurons would be pushed to fire closer to the inhibitory theta phase (Figure 2). Accordingly, advancing the firing of a subset of units could simply place those units in a phase of theta with strong LTP.

Theories of STDP [68] hold that the order of spiking determines the direction of synaptic change. In particular, a synapse increases in strength if the presynaptic unit spikes before the postsynaptic unit. This provides causality to learning; that is, earlier spike firing suggests that the presynaptic unit is contributing to driving the postsynaptic unit to fire, indicating that the synapse should increase. However, for such learning to cause sustained LTP of a synapse, a consistent pre–post order of firing needs to obtain. Thus, it could be that advancing the firing of stimulation-specific units in the hippocampus introduces a systematic order to that firing on which STDP can act.

There are forms of Hebbian learning that incorporate a normalization for the amount of weight change for each postsynaptic unit (e.g., [56], see pp. 289, 290, and 291). As a result, a bounded form of Hebbian learning is obtained, according to which weights cannot become arbitrarily large. A consequence of the normalization, however, is that competition is generated between weight changes on links entering a postsynaptic unit, with changes being bigger if fewer of these weights are subject to change. This would occur when fewer presynaptic units are active and would generate greater LTP for synapses that change when firing is selective (i.e., during an inhibitory phase). This is exactly what the advancement of spiking for stimulation-sensitive units toward the inhibitory theta phase, as we are proposing, would do: it would restrict the set of presynaptic units that are firing, ensuring greater LTP on their specific synapses. Similarly, the amount of LTD for stimulation-unspecific units would be rather small, even negligible.

## Box 2 Predictions of the Synchronization/Desynchronization Framework (SDF)

The here-presented SDF makes several predictions, which are listed below.

The level of cortical desynchronization should correlate positively with cortical information coding; that is, more desynchronization should lead to stronger fidelity of neural representations as detected with multivariate pattern-analysis methods.

During encoding, phase precession in the hippocampus should correlate with memory formation; that is, items that are later remembered should show stronger phase precession than items that are later not remembered. The theta phase should also discriminate between stimulus-selective and stimulus-unselective neurons during both encoding and retrieval.

Desynchronization in the cortex has a downstream effect on the stimulus-selective neurons in the hippocampus that should express itself in: (i) phase precession of neurons in the hippocampus; (ii) higher theta/gamma cross-frequency coupling; and (iii) higher metabolic demands [blood-oxygen-level dependent (BOLD)] signal in the hippocampus.

A relatively small proportion of hippocampal neurons should precess forward during encoding, reflecting the relatively sparse nature of hippocampal representations.

These predictions can be tested in multiple ways using various approaches and recording techniques. On a theoretical level, the predictions can be tested in computational models that combine basic ideas of CLS theory with oscillations and neural firing in the neocortex and hippocampus (see, for example, [52]). Implementing the proposed synchronization/desynchronization mechanisms in such models will show what type of behavior can be modeled and, more importantly, what type of behavior cannot be modeled. Thereby, computational modeling would reveal the limits of our framework. On an empirical basis, these predictions can be tested using invasive recording methods combining local field potential (LFP) with single-unit recordings in animal models (i.e., rodents and macaques) and humans (i.e., epilepsy patients undergoing presurgical monitoring). As the core idea of our framework relies on an interaction between the hippocampus and neocortex, it is key to not only record in the hippocampus or the neocortex but to record hippocampal and neocortical activity simultaneously. Finally, some of the predictions can also be tested using noninvasive recording methods such as EEG/MEG or EEG–fMRI. Recent developments in source localization have made it possible to record electrophysiological signals from the MTL [11]. Although it might be difficult for EEG/MEG studies to disentangle hippocampal from other activity within the MTL (e.g., parahippocampal gyrus, EC), oscillatory interactions between the MTL and the neocortex in principle can be studied. Additionally, multivariate analysis approaches using temporal and spatial pattern analysis can be utilized to test for the representation of information, which can then be linked to cortical desynchronization.

## Glossary

Complementary Learning Systems (CLS): the CLS approach was conceived as a response to the plasticity–stability dilemma, a fundamental trade-off that learning systems have to deal with. CLS proposes a means to obtain a relatively stable learning system without forfeiting plasticity and does this by positing two subsystems, the neocortex and hippocampus, with complementary representational characteristics. The neocortical system slowly learns highly ‘overlapping’ representations, with units becoming broadly tuned and thereby contributing to many different stored patterns. As a consequence, the cortical system is highly susceptible to catastrophic forgetting. The hippocampal system, by contrast, is not limited in this way. This is achieved by it exhibiting a sparse code in which the representation for each new pattern has little overlap with prior learned patterns. This pattern-separated character, which is particularly evident in the dentate gyrus and CA3, ensures that it is not subject to catastrophic forgetting in the same way as the neocortex. Furthermore, this level of overlap of units representing new and old items means that this system can learn quickly with little cost. This enables the CLS system to exhibit ‘one-shot’ learning, as we humans do. See 7, 66 for a recent review.
Desynchronization: a decrease in the neural behavior causing synchronization (see Synchronization).
Information: a very broad term with numerous different definitions. Rather than giving a further theoretical definition of information, we here refer to information in an operational way. That is, information is, for us, the degree to which we can make inferences about the identity of a particular perceived or retrieved memory from a particular observed temporal or spatial neural pattern.
Noise correlations: a plethora of single-unit recordings in monkeys has shown that even under the most controlled conditions firing rate varies significantly from trial to trial when identical stimuli are presented. Synchronized low-frequency fluctuations (<20 Hz) in firing rate, which are independent of the stimulus, are assumed to be the main driving force of this variability, termed noise correlations [49]. One way in which attention enhances the neural signal-to-noise ratio is by reducing (i.e., desynchronizing) these low-frequency fluctuations [6].
Oscillations: brain oscillations are typically divided into different frequency bands and referred to by letters of the Greek alphabet, including but not limited to delta (1–4 Hz), theta (4–8 Hz), alpha (8–12 Hz), beta (15–25 Hz), and gamma (40–80 Hz). The exact boundaries between frequencies are difficult to define and vary considerably between experiments and species.
Synchronization: there are many ways to define neural synchronization and even more methods to measure it. In this opinion article, we review studies that focused on either of the following three types of synchronization: (i) the level of synchronization within a local cell assembly, which can be measured via the power of an oscillatory signal on EEG or MEG [67]; (ii) interareal synchronization, which is the synchronization between two brain regions (e.g., the hippocampus and the rhinal cortex) [24]; and (iii) phase amplitude cross-frequency coupling, which refers to the synchronization between two frequencies whereby the phase of a lower frequency modulates the power of a higher frequency (e.g., the coupling between the phase of theta and the power of gamma 11, 30, 31).
